# Diet-induced changes in brain structure and behavior in old gerbils

**DOI:** 10.1038/nutd.2015.13

**Published:** 2015-06-15

**Authors:** K Goncharova, G Skibo, T Kovalenko, I Osadchenko, G Ushakova, M Vovchanskii, S G Pierzynowski

**Affiliations:** 1 Department of Biology, Lund University, Lund, Sweden; 2 Department of Cytology, Bogomoletz Institute of Physiology, Kiev, Ukraine; 3 Key State Laboratory, Bogomoletz Institute of Physiology, Kiev, Ukraine; 4 Department of Biophysics and Biochemistry, Oles' Honchar Dnepropetrovsk National University, Dnepropetrovsk, Ukraine; 5 Department of Theory of Random Processes, Institute of Mathematics, Kiev, Ukraine; 6 Department of Med Biology, Institute of Rural Medicine, Lublin, Poland

## Abstract

**Background/Objectives:**

Aging is associated with many physiological alterations such as changes in metabolism, food intake and brain dysfunction. Possible ways to correct age-related brain dysfunction using dietary treatments still remains undeveloped. The aim of our research was to investigate whether long-term dietary treatment with 2-oxoglutarate (2-OX), which is involved in many regulatory pathways, together with pancreatic-like enzymes of microbial origin (PLEM), which ensure appropriate digestion and absorption of nutrients, affects age-related changes in the brain morphology and cognitive function in old Mongolian gerbils.

**Materials/methods:**

Experiment was comprised of two separate studies. Samples of the hippocampus were obtained from male Mongolian gerbils of different ages (*n*=63 in the first study, *n*=74 in the second study). Immunohistochemistry was used for visualization of the nestin/NeuN-positive neuronal progenitors. Changes in amount of neural cell adhesion molecules (NCAMs) were estimated using enzyme-linked immunosorbent assay. For assessment of cognitive and sensorimotor functions, the T-maze spontaneous alternation test and the adhesive removal test (ART) were used. The ultrastructure of the CA1 hippocampal area was visualized using transmission electron microscopy.

**Results:**

Long-term treatment with 2-OX+PLEM led to a significantly increased amount of nestin/NeuN-positive cells in the CA1 hippocampal area and positive changes in learning and sensorimotor functions. As for synaptic transmission, changes in the spatial distribution of synaptic vesicles, as well as the redistribution of NCAM forms, were observed in the hippocampal synapses of the old gerbils.

**Conclusions:**

Taken together, our data show that dietary supplementation with 2-OX+PLEM not only enhances the proliferation and differentiation of neuronal progenitors, but also improves age-related deficits in the morphological and functional state of the brain of old gerbils. Thus, suggesting that a 2-OX+PLEM-enriched diet could also improve brain functions that have deteriorated with age.

## Introduction

Aging is usually associated with functional loss in many physiological systems. Humans are unique among mammals in that they are susceptible to certain neuropathologies (such as Alzheimer disease, Parkinson disease and so on) in the later stages of their life. Even in the absence of mentioned neuropathological diseases, aging in humans is marked by variable degrees of neuronal deterioration and cognitive impairment. One of the reasons for the development of cognitive disorders during aging is the insufficient digestion and assimilation of nutrients in the elderly. The hippocampus is one of the brain regions that undergoes substantial age-related changes.^1^ The morphological changes probably make a significant contribution to the behavioral impairments and cognitive decline that often accompany aging.^2^

Research has demonstrated that nutrients, which have high antioxidant activity, can have beneficial cognitive anti-aging effects. For example, feeding aged rats with a diet rich in blueberries improves their water-maze performance^3^ and hippocampal plasticity parameters.^4^ Patients with dementia display reduced plasma antioxidant levels^5^ and dietary antioxidants appear to protect them against cognitive impairment.^6^ 2-Oxoglutarate (2-OX) is a well-known intermediate product of the Krebs cycle. 2-OX has been studied for decades due to its anticarcinogenic abilities.^7^ Blood levels of 2-OX can be dependent on intestinal levels of 2-OX, as 2-OX transporters of high affinity and low capacity often occur along the entire gastrointestinal tract.^8, 9^ Thus, the 2-OX originating from the gut could be important in maintaining metabolic homeostasis throughout the body, including within the brain, as it has been recognized as a strong protector of neurons against specific noxious substances.^10^ Finally, our studies have shown that dietary 2-OX has the capacity to improve blood circulation within the brain and ameliorate the consequences of ischemic stroke.^11^

Aging is associated with profound changes in nutrient digestion and absorption.^12^ At the same time, pancreatic function is also impaired.^13^ It is also well known that the elderly suffer from enzyme insufficiency and often have maldigestion and malabsorption.^12^

Hypothetically, beneficial nutrients ingested by the elderly could significantly influence brain function. However, the lack of appropriate gastrointestinal tract functions could nullify the effects of food components on cognition. Thus, we hypothesized that dietary supplementation of pancreatic-like enzymes of microbial origin (PLEM) could improve brain function, through the enhancement of digestion and absorption of beneficial dietary compounds.

The aim of our study was to investigate whether long-term dietary treatment with 2-OX (a gut bacterial product) and PLEM (replacement therapy for the malfunctioning pancreas) affects age-related changes in brain morphology and cognitive function of old Mongolian gerbils (old MGs)—animals that represent a well-known model of neurodegenerative disorders and behavioral studies.^14^

## Materials and methods

### Animals and housing

The study was carried out at the Animal house of Bogomoletz Institute of Physiology, National Academy of Science of Ukraine (Kiev), in strict accordance with the recommendations in the Guide for the Care and Use of Laboratory Animals of the National Institute of Health. All experimental procedures were approved by the Local Animal Bioethics Committee of Bogomoletz Institute of Physiology, located in Kiev, Bogomoletz str., 4.

Old male Mongolian gerbils aged between 1.5–2 years old at the start of the experiment and 2–2.5 years old at the end of the experiment were used in the study. Young male gerbils aged 6 months were used for comparison as an additional ‘age-control'. Gerbils were housed in standard cages (48 × 27 × 20 cm) with free access to food and tap water (2–6 gerbils per cage). Gerbils were kept in a temperature-controlled room (22±2 °C), under a constant 12:12-h light/dark cycle (lights on at 0600 hours).

### Feeding of animals and administration of 2-OX and PLEM

Food and water were provided *ad libitum* before and during the experimental period. Four different types of dry food were produced for the study (Morawski Feed Plant, Poland). The standard diet composition is provided in Table 1. The first diet served as the standard diet, the second diet was enriched with 2-OX (10 g of Ca 2-OX (Rexim, Ham, France) and 10 g of Na_2_ 2-OX (Rexim) per 1 kg of food) and the third diet was enriched with PLEM (726 000 units per kg feed of lipase (*Burkholderia cepacia*), 486 000 units per kg feed of protease (*Aspergillus melleus*) and 60 000 units per kg feed amylase (*Aspergillus oryzae*)). To the fourth diet, both 2-OX and PLEM were added. PLEM as well as 2-OX were added in the same amounts as in the second and third diets during manufacture and further analyses showed a recovery of between 80 and 90%. All enzymes were provided by Amano Enzyme, Inc., Japan.

### Experimental procedures

#### Our research included two studies (study 1—S1 and study 2—S2)

For study 1 (S1), 63-year-old male MGs were used. The MGs were randomized into four groups—control old MGs, which received the standard diet (old control group, *n*=18), old MGs, which received 2-OX as an addition to the diet (2-OX-diet group, *n*=14), old MGs, which received PLEM (PLEM-diet group, *n*=14), and old MGs, which received both 2-OX and PLEM (2-OX+PLEM group, *n*=17). Six young male MGs (6 months of age) that received the standard diet were used as an additional ‘age-control' (young control group, *n*=6). All animals received their respective diets for a period of 6 months. At the end of the treatment period, cognitive function of the MGs was assessed using the T-maze spontaneous alternation test. Half of the gerbils from each group were then anaesthetized and transcardially perfused for morphological analyses. The other half of the MGs were then killed by decapitation for biochemical analyses.

Study 2 was performed to estimate the behavioral parameters of the old MGs (72 animals) during the long-term dietary administration of 2-OX+PLEM. The MGs were randomized into two groups, the first group (2-OX+PLEM-diet, *n*=37) received the 2-OX+PLEM-enriched diet and the second group of MGs (old control, *n*=35) received the standard diet. All gerbils received their respective diets for a period of 5 months; as following on from S1, we assumed this duration to be sufficient to influence brain morphology and function. During the 5-month treatment period, the MGs' behavior was analyzed using the T-maze spontaneous alternation and ART. The T-maze spontaneous alternation measurement and ART were performed four times (each test comprised of three repetitions, thus 12 trials in total) during the 5-month treatment period.

### Behavior assessment

#### T-maze spontaneous alternation

Spontaneous alternation measures an animal's ability to remember which arm in a T-maze it had previously entered and thus allowing the animal to explore alternate arms of the maze in repeated trials.^15, 16^ Gerbils were placed in the start chamber of a transparent, acrylic, T-maze. The protocol for spontaneous alternation testing began with a gerbil that was confined to the start chamber for 5 s and then permitted access to the rest of the maze for 3 min. There were two phases to each trial: a sample phase where the animal runs to one goal arm of the maze and a choice phase where the animal chooses another arm of the maze.^17^ The percentage of alternation (number of turns in each goal arm/total number of turns) was recorded.

#### Adhesive removal test

ART was adapted from studies in rats^18^ to investigate sensorimotor function in the MGs. Adhesive dots (0.6-cm diameter, Avery) were placed on both forelimbs of the gerbils, after which they were returned to their cages. The time it took for the gerbils to remove the dot from each forelimb was recorded. If a gerbil did not remove one of the adhesive dots on either the right or left forelimb, or both stickers within 180 s, the gerbil received a score of 180 (seconds) for the respective forelimb(s).

### Immunohistochemistry

MGs were anaesthetized with ketamine (100 mg kg^−1^ body weight i/m) and fixed by intracardial perfusion with 4% formaldehyde and 0.25% glutaraldehyde in 0.1 m phosphate buffer. After perfusion, the brains were isolated and dissected into two hemispheres. Samples for immunohistochemistry (one hemisphere from each gerbil) were postfixed overnight in the same fixative at +4 °C. Goat monoclonal anti-nestin antibodies (1:500; Santa Cruz Biotechnology Inc, Dallas, TX, USA) and mouse anti-NeuN antibodies (diluted 1:1000; Merck Millipore, Billerica, MA, USA) were used for detection of immature neuronal cells. Slices were incubated with primary antibodies for 16 h at +4 ^o^C and then with secondary antibodies, anti-mouse conjugated with Alexa Fluor 555 (1:1000) and anti-goat conjugated with Alexa Fluor 647 (1:1000) for 1.5 h at room temperature (Molecular Probes, Eugene, OR, USA). For each experimental and control group, 10 randomly selected slices per gerbil were analyzed.

### Electron microscopy

The hippocampus from the other brain hemisphere of each gerbil was prepared and embedded in EPON resin, according to the standard protocol.^19^ Ultrathin sections (70 nm) from the middle portion of the CA1 stratum pyramidale and stratum radiatum were stained with uranyl acetate and lead citrate. The images were obtained using a JEM-100CX (Jeol, Tokyo, Japan) transmission electron microscope at a magnification of × 10 000. The shortest distance from a synaptic vesicle (SV) to the active zone profile (active zone distance (AZD)), as well as the spatial proximity of the SV profiles to each other (nearest neighbor distance (NND)) were quantified using LoClust software.^20^ For each experimental and control group, 100 synapses containing SVs were analyzed.

### Determination of NCAM level by enzyme-linked immunosorbent assay

After decapitation of the gerbils, the brains were quickly dissected out and the hippocampi were isolated and immediately homogenized in 10 volumes of 25 mm Tris-HCl buffer, pH7.4, containing 1 mm EDTA, 2 mm dithiothreitol, 0.2 mm phenylmethanesulfonyl fluoride and 0.01% merthiolate, at +4 ^o^C. The levels of neural cell adhesion molecules (NCAMs) in the hippocampal tissue were measured using a non-competitive enzyme-linked immunosorbent assay. Optical density was measured using an Anthos-2010 absorbance reader (Anthos Labtec Instruments, Wals-Siezenheim, Austria).^21^

### Statistical analysis

Statistical analysis was performed using Statistica software (version 7, StatSoft, Tulsa, OK, USA). Values are shown as mean±s.d. Distribution of the groups was analyzed using the Kolmogorov–Smirnov test. A Two-way analysis of variance and Tukey *post hoc* test were used to assess the differences between experimental groups (*P*⩽0.05 was considered to indicate statistical significance).

## Results

### Morphology of the CA1 hippocampal area of MGs

**Figure 1:**
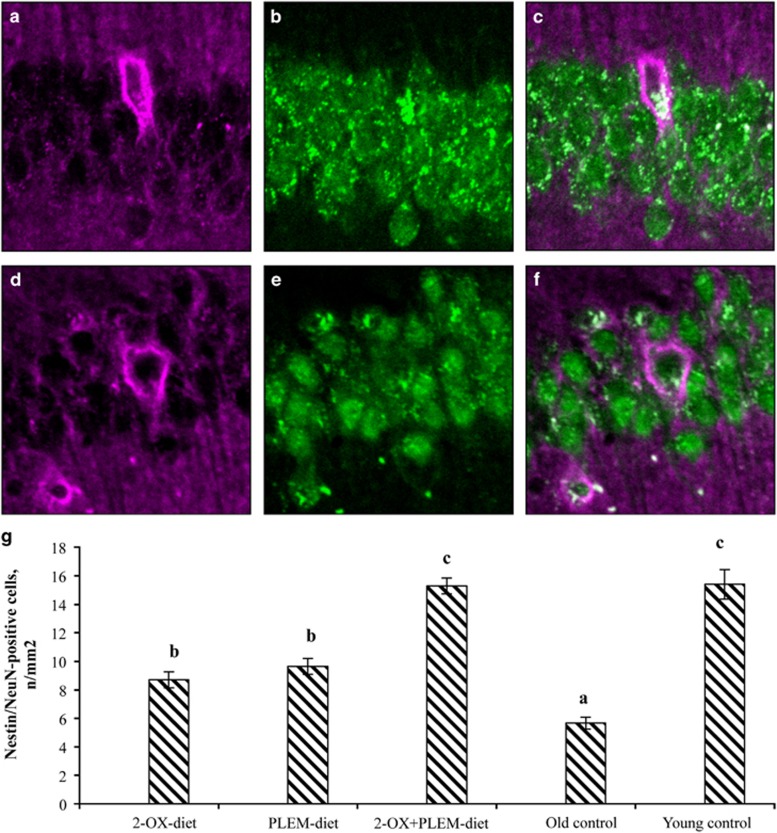
Immunohistochemical analysis of hippocampal CA1 area. (**a**–**f**) Consistency of nestin (violet) and NeuN (green) staining in the CA1 hippocampal area of MGs. (**a**–**c**) Old control group. (**d**–**f**) 2-OX+PLEM group. (**a** and **d**) Nestin-positive cells (purple). (**b** and **e**) NeuN-positive cells (green), (**c** and **f**) merged. (**g**) Number of Nestin/NeuN-positive cells per 1 mm^2^ of stratum pyramidale in hippocampal CA1 area. 2-OX-diet group—old MGs treated with 2-OX-enriched diet (*n*=14); PLEM-diet group—old MGs treated with PLEM-enriched diet (*n*=14); 2-OX+PLEM group—old MGs treated with both 2-OX and PLEM (*n*=17); old control group—control old MGs (*n*=18); young control group—young MGs (6 months; *n*=6). Data presented as mean±s.d. ^a,b,c^Mean values with unlike letters were significantly different between the groups (*P⩽*0.05).

The CA1 hippocampal area was chosen for our investigation as one of the brain regions most affected by aging.^22^ On the basis of the different patterns of pyramidal neuron staining, two major subpopulations of these cells were recognized. One type of pyramidal neurons was only NeuN-positive, whereas the second type revealed both NeuN and nestin-positive staining. The number of nestin/NeuN-positive neurons was much less than the amount of NeuN-positive cells and significantly different between experimental groups (Figure 1a and f).

Nestin/NeuN-positive cells were revealed in the stratum pyramidale of the hippocampus. In some cells, long processes that seem to be dendrites extended into deeper layers of the hippocampus, and these processes were also nestin positive. A significantly higher amount of nestin/NeuN-positive cells was found in gerbils in the young control group, compared with those of the old control group (*P*=0.000001). The number of nestin/NeuN-positive cells in the CA1 hippocampal area of old gerbils was also significantly increased after long-term dietary treatment (in groups 2-OX-diet, PLEM-diet (*P*=0.008 and *P*=0.000023, respectively) and 2-OX+PLEM-diet; *P*=0.000001; Figure 1g).

### Ultrastructure of the CA1 hippocampal area of MGs

In the middle portion of the CA1 stratum radiatum analyzed in the study, the great majority of synaptic inputs are excitatory and they preferentially terminate on the dendritic shaft. Taking these aspects into consideration, we could focus our analyses on the pool of excitatory CA1 spine synapses.

**Figure 2:**
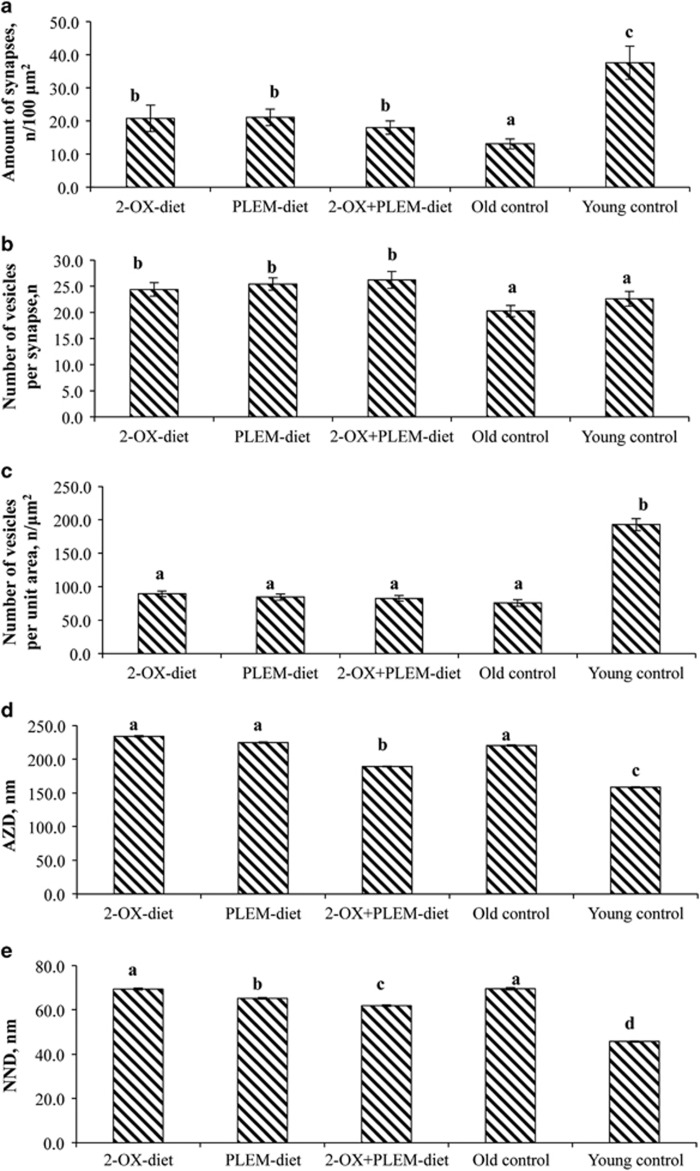
Ultrastructure of the CA1 hippocampal area of MGs. (**a**) Number of synaptic terminals per unit area of hippocampal CA1 zone. (**b** and **c**) Number of SVs per synapse (**b**) and per 100 μm^2^ of synaptic terminal (**c**) in hippocampal CA1 zone. (**d**) Distance from synaptic vesicles to the active zone (AZD) of the presynaptic terminal. (**e**) Distance from vesicle to the nearest neighbor (NND) vesicle of presynaptic terminal. 2-OX-diet group—old MGs treated with 2-OX-enriched diet (*n*=14); PLEM-diet group—old MGs treated with PLEM-enriched diet (*n*=14); 2-OX+PLEM group—old MGs treated both with 2-OX and PLEM (*n*=17); old control group—control old MGs (*n*=18); young control group—young MGs (6 months; *n*=6). Data presented as mean±s.d. ^a,b,c^Mean values with unlike letters were significantly different between the groups (*P⩽*0.05).

The number of synaptic terminals per unit area (100 μm^2^) in the old control group was three times lower compared with that of the young control group, but after long-term dietary supplementation with 2-OX and PLEM the number of synaptic contacts increased to 155% (groups 2-OX-diet and PLEM-diet groups) and 147% (2-OX+PLEM-diet group; Figure 2a).

The number of SVs as well as their spatial arrangement can characterize neurotransmitter turnover. Numbers of SVs in the 2-OX-diet, PLEM-diet and 2-OX+PLEM-diet groups were not significantly different and were higher than those of the old control and young control groups. The results of the statistical analysis indicated significant differences for the 2-OX-diet, PLEM-diet and 2-OX+PLEM-diet groups in comparison with both the old control (*P*=0.035; *P*=0.047 and *P*=0.023, respectively) and young control groups (*P*=0.029; *P*=0.040 and *P*=0.037, respectively). At the same time, the numbers of SVs were not significantly different between old control and young control groups (Figure 2b). In addition, no significant differences in the number of SVs per square units (100 μm^2^) of terminal (NVT) were observed between the 2-OX-diet, PLEM-diet and 2-OX+PLEM-diet groups of MGs (Figure 2c).

All groups of old MGs (both treatment groups and the old control group) had significantly increased AZD compared with the gerbils from the young control group. But at the same time, a significant decrease in AZD in the 2-OX+PLEM-diet group (*P*=0.045) compared with the old control group was observed, whereas the differences in the 2-OX-diet and PLEM-diet groups failed to reach significance compared with old control group (Figure 2d). Such a decrease in AZD value can result in the redistribution of SVs and the improvement of synaptic transmission velocity.

Distance to the first NND of each vesicle was used to measure the tendency of SVs to form spatial clusters. The analysis of vesicle clustering showed that SVs were also separated by a larger distance in aged MGs: the average NND value estimated for old control MGs was ~30% higher than that of the young control MGs (Figure 2e). The results of the statistical analysis revealed a significant increase in NND for the 2-OX-diet, PLEM-diet, 2-OX+PLEM-diet and old control groups of MGs compared with the young control group (*P*=0.035; *P*=0.046; *P*=0,043; *P*=0.038, respectively). It is worth noticing that in the 2-OX+PLEM-diet group, the NND was significantly different from values observed for the 2-OX-diet, PLEM-diet and old control groups (*P*=0.038; *P*=0.047; *P*=0.023, respectively) and closer to the values observed in gerbils from the young control group.

### Levels of NCAM in the hippocampus of MGs

**Figure 3:**
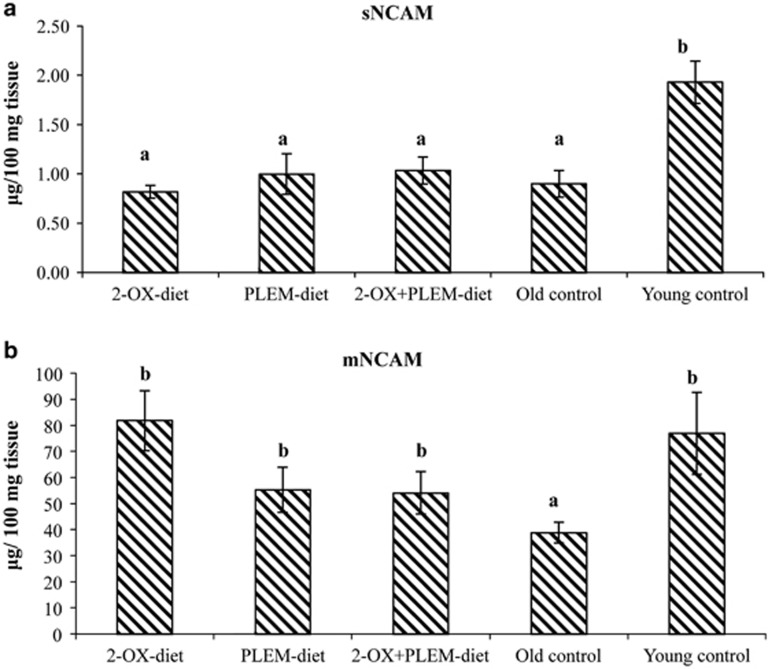
The distribution of soluble (**a**) and membrane (**b**) forms of NCAM of gerbil hippocampus. 2-OX-diet group—old MGs treated with 2-OX-enriched diet (*n*=14); PLEM-diet group—old MGs treated with PLEM-enriched diet (*n*=14); 2-OX+PLEM group—old MGs treated both with 2-OX and PLEM (*n*=17); old control group—control old MGs (*n*=18); young control group—young MGs (6 months; *n*=6). Data presented as mean±s.d. ^a,b,c^Mean values with unlike letters were significantly different between the groups (*P*⩽0.05).

Two different subcellular protein fractions (water-soluble/cytosolic and membrane fractions) were obtained using differential ultracentrifugation of dissected hippocampi, to study the distribution of soluble and membrane forms of NCAM. The level of sNCAM was decreased in the hippocampus of old MGs from the 2-OX-diet, PLEM-diet, 2-OX+PLEM-diet and old control groups, compared with the young control group of MGs (Figure 3). However, the differences in the level of total proteins in the water-soluble fraction extracted from the hippocampus were not significant. The level of membrane NCAM was significantly (*P*=0.034) decreased in the old control group of MGs compared with the young control group of MGs. Long-term dietary supplementation of old gerbils with 2-OX and PLEM prevented such a large decrease in membrane NCAM in the hippocampus. The level of mNCAM in the hippocampus of old gerbils receiving the 2-OX-diet, PLEM-diet and 2-OX+PLEM-diet was significantly higher (*P*=0.043, *P*=0.024 and *P*=0.039, respectively) than that observed in the old control group of MGs.

### MGs' behavioral assessment

#### T-maze spontaneous alternation test

The T-maze spontaneous alternation measurement test, which was performed on the MGs once (test comprised of three repetitions) at the end of S1, revealed a tendency for decreased rates of correct alternation in all groups of old MGs (<70%), compared with the young control group. The highest rates of alternation (>80%) were observed in the young control group. In the PLEM-diet and 2-OX+PLEM-diet groups, the percentage of correct alternation was ~75% (data not shown).

**Figure 4:**
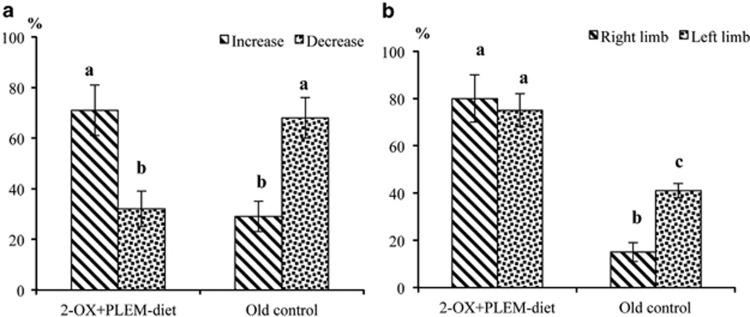
(**a** and **b**) Behavioral assessment of MGs in study 2. (**a**) The percentage of old Mongolian gerbils that demonstrated positive (increase) or negative (decrease) trends in the T-maze spontaneous alternation test during the study. (**b**) The ratio of old Mongolian gerbils that improved their performance in the ART for left and right limbs during the study. 2-OX+PLEM-diet group—old MGs treated with both 2-OX and PLEM (*n*=37); old control group—control old MGs (*n*=35). ^a,b,c^Mean values with unlike letters were significantly different between the groups (*P⩽*0.05).

The differences in the gerbils' behavior observed at the end of S1 were intriguing and we decided to perform the second study for precise behavior estimation. For the second study, only the effects of 2-OX+PLEM treatment were investigated because the 2-OX+PLEM-diet group demonstrated maximal improvement of morphological parameters during S1. During study 2, the T-maze spontaneous alternation measurement test was performed four times (each test comprised of three repetitions) during the 5-month treatment period. During the first 4 months of treatment, we observed a continuous improvement in spontaneous alternation level in gerbils from the 2-OX+PLEM-diet group. At the same time, for gerbils in the old control group, the alternation level was not significantly different between the time points. To characterize the ability of the MGs to improve the spontaneous alternation level during the study, the difference between the percentage of tasks solved in the first trial and the mean of the percentages of tasks solved in subsequent trials were used. A positive difference (increase) was interpreted as an increase in ability to pass the test. The difference was calculated for each gerbil, in both the 2-OX+PLEM-diet group and the old control group. The results of the analysis of variance indicated a significant difference (*P*=0.037) between experimental groups. The gerbils in the 2-OX+PLEM-diet group demonstrated an increase in the number of tasks solved per test, more often than the gerbils in the old control group. The MGs that received the 2-OX+PLEM-diet demonstrated an increase in the number of tasks solved per test in 68% of cases compared with the 29% increase achieved by the old control group (Figure 4a).

#### Adhesive removal test

The ART test was repeated four times (three repetitions per trial) during the 5-month treatment period, each trial resulting in two measurements: the time it took for the MGs to remove an adhesive dot from the right limb and then from the left limb, respectively. The individual tendency of gerbils to improve their test performance was assessed. Trials in which the gerbils had not removed an adhesive dot in 180 s were omitted from the investigation. In the 2-OX+PLEM-diet group, 79% (‘right limb') and 72% (‘left limb') of MGs showed an improvement in the task performance, which meant the time it took to pass the test declined over time for most rodents in the group. MGs in the old control group only achieved an improvement of 13% and 40%, respectively, which may naturally be interpreted as the old rodents that did not receive 2-OX+PLEM treatment found the test task more difficult (Figure 4b). The results of the analysis of variance indicate a significant difference (*P*⩽0.025) between experimental groups.

## Discussion

Aging is associated with cognitive impairments, many of which involve damage to the amygdala and hippocampus.^23^ Gerbils are well-known models for neurophysiology, they mature at the age of 5 months and their lifespan is around 2.5–3 years.^24^ Our present research has shown that the learning and memory abilities of MGs decreases with aging (S1) and that enrichment of the gerbils' diet with 2-OX and PLEM can increase the cognitive function parameters of aged MGs. Old MGs that did not receive dietary treatment tended to show worse results over time. Long-term dietary treatment with 2-OX+PLEM markedly reduced errors in the performance of both behavioral tasks by aged MGs, indicating that 2-OX and PLEM, given in combination, can improve learning memory deficits in old MGs.

An increasing number of reports have suggested that hippocampal neurogenesis is involved in learning and memory.^25, 26^ Li *et al.*^27^ has shown that aging animals who display normal or even higher levels of nestin-positive cells in the hippocampus and a normal number of NeuN+ cells maintain their cognitive function. In our study, nestin-positive cells were found in the layer of pyramidal neurons, these cells revealed strong NeuN staining. The structural similarities between nestin/NeuN-positive cells and neurons, allow us to consider these types of cells as newly generated neurons. The largest number of nestin/NeuN-positive cells was found in the group of young gerbils, in comparison with the control group of old gerbils. The amount of nestin/NeuN-positive cells in the samples from experimental groups increased after long-term dietary treatment (2-OX-diet, PLEM-diet and 2-OX+PLEM-diet groups). Thus, our data confirm previous findings, which suggest that neurogenesis is remarkably impaired by aging, perhaps due to the decrease in the number of neural stem cells present in the aged hippocampus^28, 29^ and at the same time allow us to suggest the enhancing influence of long-term dietary treatment with 2-OX and PLEM on the process of neurogenesis in old gerbils.

Drapeau *et al.*^30^ reported a decreased number of hippocampal neurons in aged animals that was associated with a decline in their spatial learning and memory abilities. As the hippocampus is involved in the learning and memory processes,^1, 31^ in our present study, we tried to elucidate the changes in hippocampal synapses in MGs of increasing age. If the number of synapses decreases, the plasticity and transmission of synapses are weakened, and in turn the learning and memory abilities are affected.^32, 33, 34^ The CA1 hippocampal area of old control gerbils exhibited the lowest synapse count, as well as the lowest number of vesicles per synapse as compared with the 2-OX- and/or PLEM-treated experimental groups. The redistribution of SVs inside the synaptic terminal (the highest AZD and NND) that was observed in the old control group of MGs also indicates weak inter-neuronal connections. Supplementation of the diet with 2-OX+PLEM significantly reversed these changes and made the synaptic parameter values closer to those observed in the young control group gerbils. Furthermore, we infer that one of the mechanisms by which 2-OX protects synapses may be related to its antioxidant activity, because oxidative stress has an effect on neuron uptake and retention of glutamate, and induces excitotoxic neuronal death.^35^

Many of the NCAMs have demonstrated a positive influence on the synaptic plasticity in animal models.^36, 37^ NCAM regulates synapse formation, maturation and function through homo- and heterophilic interactions.^38^ The ablation of NCAM reduces the number of synapses.^39^ The C-terminal of NCAM has a key role in maintaining effective transmission via a pathway involving myosin light chain kinase and probably MLC and myosin II.^40^ Our data have demonstrated that NCAM expression in the gerbil's hippocampus appeared to decrease in aged animals compared with young MGs, reflecting synaptic elimination, as has been demonstrated in old animals after ultrastructural analyses. The results obtained showed a positive effect of the 2-OX- and PLEM-enriched diet in the old gerbils, in the prevention of a decrease in membrane NCAM level in the hippocampus.

Previous studies in our lab have shown the protective and anti-degenerative effects of dietary 2-OX during ischemic brain injury.^11^ In the elderly, 2-OX blood levels are decreased and this reduction could be explained by both reduced metabolism and changes in gut microbiota, which could serve as a source of 2-OX in the GIT of humans.^41^ 2-OX has been shown to inhibit oxidative stress induced by H_2_O_2_ in erythrocytes and cultured neurons.^42, 43, 44^ Moreover, 2-OX was shown to modulate the activity of antioxidant enzymes and stabilize redox homeostasis in older mice to the level observed in young animals.^45^ 2-OX also participates in many pathways of homeostatic regulation. Recently, Chin *et al.*^46^ demonstrated that 2-OX extends the lifespan in both *Caenorabditis elegans* and mammalian cells and provided evidence that the lifespan increase by 2-OX is dependent on the target of rapamycin, downstream pathway. As the target of rapamycin pathway has an essential role in numerous key cellular processes, its deregulation is implicated in various diseases such as cancer, diabetes and neurodegeneration.^47^ In addition, dietary supplementation with 2-OX has been shown to alleviate intestinal injury and improve intestinal absorption.^48, 49^ Thus, one can speculate that the neuroprotective effects of 2-OX as a dietary supplement are realized both through the changes in homeostatic regulation and improved absorption in small intestine.

At the same time, a number of structural changes have been described in the aging pancreas. These changes have been ascribed to pancreatic involution leading to a decreased secretory capacity of the exocrine pancreatic enzymes.^50^ Thus, the resulting age-related exocrine pancreatic insufficiency makes the absorption of most nutrients less effective, especially that of long-chain polyunsaturated fatty acids (PUFAs), which are crucial nutritional components for brain plasticity. Thus, one can speculate that cognitive function and some parameters of hippocampal plasticity were improved by the increased level of 2-OX and PUFA in the blood of old MGs after long-term dietary supplementation with 2-OX and pancreatic-like enzymes. Data from our lab obtained using pigs have partially proven this hypothesis. PUFA levels in the plasma and other tissues were lower in an exocrine pancreas insufficient pig model and increased after PLEM dietary supplementation.^51^ The dietary supplementation with 2-OX enhanced plasma levels of 2-OX^52, 53^ and alternated energy metabolism in the pigs.^54^

The combined effects of long-term dietary treatment with both 2-OX and PLEM can be realized due to different pathways of neuroprotection provided by these compounds. Our results suggest that the combined long-term dietary treatment with 2-OX and PLEM has additive neuroprotective properties. A combination of the prevention of oxidative stress, together with an improvement in the absorption of necessary nutrients leads to the amelioration of age-related cognitive dysfunction and degenerative changes in synaptic morphology. In conclusion, our data allow us to suggest that a diet enriched 2-OX and PLEM could be useful in protection of the elderly against age-related brain damage.

## Figures and Tables

**Table 1:** Standard diet composition (in diets with 2-OX supplementation the amount of starch was proportionally reduced)

*Compound*	*Amount, g kg^−1^ dry weight*	*Compound*	*Amount, g kg^−1^ dry weight*
Protein	243.056	Iodine	0.003
Fat	44.444	Copper	0.018
Fiber	88.889	Iron	0.207
Calcium	12.847	Sulfur	2.222
Phosphorus	9.486	Zink	0.107
Sodium	2.806	Cobalt	0.002
Starch	458.333	Selenium	0.005
Lysine	12.528	Vitamin A	12 000 units
Methionine	3.750	Vitamin D3	800 units
Methionone+Cysteine	8.611	Vitamin E	0.108
Tryptophan	3.333	Vitamin K3	0.003
Threonine	8.861	Vitamin B1	0.014
Arginine	15.278	Vitamin B2	0.008
Histidine	6.236	Vitamin B6	0.017
Leucine	17.500	Vitamin B12	0.000
Isoleucine	9.931	Biotin	0.001
Valine	11.931	Folic acid	0.003
Phenilalanine+Thyrosine	19.167	Nicotinic acid	0.157
Magnesium	3.972	Pantothenic acid	0.036
Potassium	13.250	Choline	2.747
Manganese	0.104	Linoleic acid	0.013
